# The Role of Hormones and Trophic Factors as Components of Preservation Solutions in Protection of Renal Function before Transplantation: A Review of the Literature

**DOI:** 10.3390/molecules25092185

**Published:** 2020-05-07

**Authors:** Aneta Ostróżka-Cieślik, Barbara Dolińska

**Affiliations:** 1 Department of Pharmaceutical Technology, Faculty of Pharmaceutical Sciences in Sosnowiec, Medical University of Silesia, Kasztanowa 3, 41-200 Sosnowiec, Poland; bdolinska@sum.edu.pl; 2 “Biochefa” Pharmaceutical Research and Production Plant, Kasztanowa 3, 41-200 Sosnowiec, Poland

**Keywords:** renal transplantation, hormones, trophic factors, organ preservation solution

## Abstract

Transplantation is currently a routine method for treating end-stage organ failure. In recent years, there has been some progress in the development of an optimal composition of organ preservation solutions, improving the vital functions of the organ and allowing to extend its storage period until implantation into the recipient. Optimizations are mostly based on commercial solutions, routinely used to store grafts intended for transplantation. The paper reviews hormones with a potential nephroprotective effect, which were used to modify the composition of renal perfusion and preservation solutions. Their effectiveness as ingredients of preservation solutions was analysed based on a literature review. Hormones and trophic factors are innovative preservation solution supplements. They have a pleiotropic effect and affect normal renal function. The expression of receptors for melatonin, prolactin, thyrotropin, corticotropin, prostaglandin E1 and trophic factors was confirmed in the kidneys, which suggests that they are a promising therapeutic target for renal IR (ischemia-reperfusion) injury. They can have anti-inflammatory, antioxidant and anti-apoptotic effects, limiting IR injury.

## 1. Introduction

Transplantation is one of the methods for treating end-stage renal failure. Compared to dialysis, it significantly improves the patient’s quality of life and reduces the cost of treatment. The number of transplanted kidneys is growing every year, as is the number of people waiting for a transplant. That is why it is important to conduct research aimed at developing preservation solutions allowing for the longest possible organ storage outside the donor’s organism and minimizing ischemia-reperfusion injury of isolated grafts. It is also crucial to extend the criteria for selecting donors to enable the collection of marginal organs and increase the number of organs available for transplantation.

Recently, a lot of research has been devoted to interference into the composition of commonly used preservation solutions aimed at their optimization. The subject of the study are biologically active compounds that, when added to solutions, can minimize transplant-related complications. These include hormones, trophic factors, minocycline (p38MAPK inhibitor), antioxidants (vitamin C, vitamin E, *N*-acetylcysteine, coenzyme Q10), micronutrients (zinc, selenium), which can potentially affect organ regeneration [1,2,3]. Numerous studies confirm the protective effect of hormones and trophic factors on the ischemic kidney. It has been found that they can have anti-inflammatory, antioxidant and anti-apoptotic effects, limiting IR (ischemia-reperfusion) injury. Hormones counteract oxidative stress, compensate for metabolic disorders of the ischemic kidney and/or stabilize its cell membranes. Clinical and pre-clinical studies indicate their relationship with the proper functioning of stored grafts [4,5].

The purpose of the review was to analyse the effectiveness of hormones as components of renal perfusion and preservation solutions. The analysis was based on the effectiveness of modified solutions in minimizing ischemia-reperfusion injury during renal transplantation.

## 2. Literature Search

The analysis covers papers in English and Polish from the last 25 years (1995–2020). Two authors independently searched the Medline electronic database (Pubmed), Cochrane Library and Google Scholar for articles on the effectiveness of modifying renal perfusion and preservation solutions with hormones and trophic factors, and published until March 1, 2020. Each article was assessed based on structured assessment tools. The articles related to studies using human and animal kidney perfusion and preservation models, which considered each animal species, age, sex, race and sample size, were included. The studies in which hormones and trophic factors were administered to the human or animal and/or directly into the kidney at any stage of the experiment in the form of injection and/or infusion or in the diet were excluded. The terms from the Medical Subject Headings (MeSH) list, i.e., organ preservation solutions, therapeutic use, hormones, additives, pharmacological agent, trophic factors, perfusion, ischemia reperfusion, kidney/renal transplantation, fatty kidney/renal models, were searched with logical operators (AND, OR, NOT). Publications were also searched manually, including review articles, meta-analyses and conference summaries, in order to identify potential studies corresponding to the adopted search criteria. Initially, the literature review comprised 425 articles. After applying the inclusion/exclusion criteria, 15 articles remained for the analysis.

## 3. Renal Ischemia-Reperfusion Injury

Ischemia-reperfusion injury (IRI) occurring during renal transplantation is unavoidable and significantly affects subsequent transplant functions, especially in the case of expanded criteria donors (ECD). The phases of graft injury can be divided into periods of warm ischemia during organ collection, cold ischemia during hypothermic storage, and organ reperfusion in the recipient. During warm ischaemia time (WIT), organ blood flow stops. Lack of oxygen and high-energy ATP compounds activates cytotoxic enzymes and generates irreversible changes. There is the transition from aerobic to anaerobic metabolism [6,7,8]. The Bowman capsule contracts and the glomerular filtration decreases [9,10]. Morphological changes occur in the distal and collecting tubules of kidney cortex. The endothelium is damaged and blood cells linger in the intravascular area [11]. Acid-base homeostasis at renal tubules is disturbed. There is a change in the activity of membrane transporters of renal tubular epithelial cells responsible for the regulation of intracellular pH and reabsorption of bicarbonates [12,13,14].

During cold ischemia time, the organ is usually stored by simple hypothermia (SCS, simple cold storage) or constant perfusion through the renal vessels using a pump. Hypothermic machine perfusion (HMP) is most commonly used for kidneys from expanded criteria donors and donors after cardiac arrest. The kidneys are cooled to +4 °C, rinsed, and then stored in a cold preservation solution until their implantation into the recipients [15,16]. Ischemic injury deepens during preservation and its severity depends on the applied storage method, solution and ischemic time. In hypothermia, renal filtration decreases proportionally with decreasing temperature. The oxidative phosphorylation process in mitochondria decreases and oxygen consumption falls. Sodium-potassium and calcium-magnesium pumps become inefficient, which results in cell oedema. The concentration of lactates and hydrogen ions increases, which acidifies the intracellular environment. The synthesis of ATP compounds is inhibited. Phosphate residues become detached, resulting in the formation of adenosine, a source of hypoxanthine that accumulates in the cell. Xanthine dehydrogenase, which acts as an oxidase, i.e., oxidizes hypoxanthine to xanthine and uric acid, undergoes transformation. Free superoxide radicals are generated. They damage the structure of structural proteins and enzymes and induce the production of inflammatory mediators. The cell membrane is destabilized as a result of lipid peroxidation [14,17,18,19].

During the implantation period, the organ gradually warms up. Oxygen demand and ischemic damage increase. ATP catabolism induces further hypoxanthine accumulation and ROS formation. These in turn prolong the expression of P-selectin, which activates neutrophils. Cytokines and proteases are released. Capillary patency is impaired. Free oxygen radicals cause damage to membrane structures, cellular proteins, cell membrane lipids and nucleic acids. There is increased cytokine production [6,20]. Intensive ROS production is observed in epithelial cells of the proximal tubules of the nephron [21]. As a consequence, the cytoskeleton is damaged, microvilli disappear, mitochondrial structures break, renal tubular epithelial cell necrosis and endothelial cell necrosis appear. The brush border is damaged and cells become detached from the basement membrane [22].

**Figure 1:**
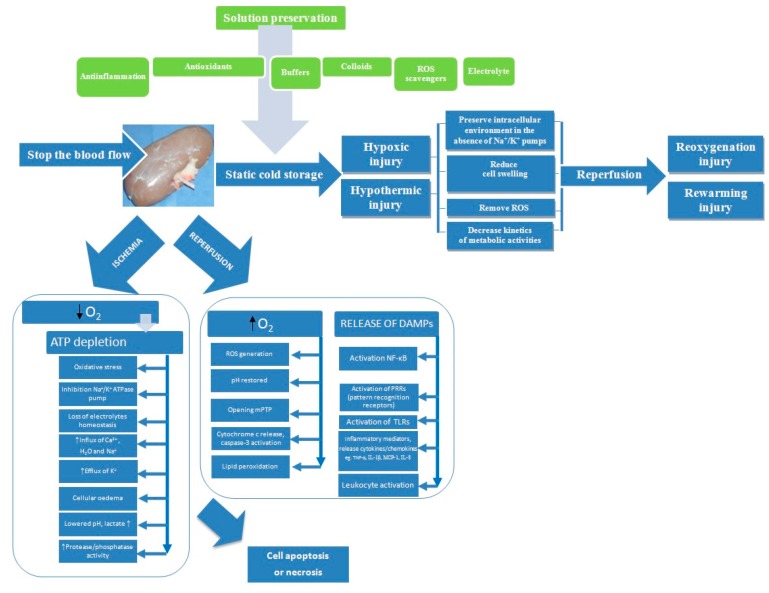
Processes involved in kidney preservation and ischemia/reperfusion injury. ATP, adenosine triphosphate; DAMPs, damage associated molecular patterns; IL-1β, interleukin 1 beta; IL-8, interleukin-8; MCP-1, monocyte chemoattractant protein-1; mPTP, mitochondrial permeability membrane transition pore; NF-κB, nuclear factor kappa B; ROS, reactive oxygen species; TNF-α, tumor necrosis factor alpha; TLRs, toll-like receptors.

Zhao et al. [19] suggested that early IRI (Ischemia-Reperfusion Injury) contributes to later graft loss as a result of reduction of renal functional mass, graft vascular injury, chronic hypoxia, and subsequent fibrosis. Renal perfusion and preservation solutions can minimize ischemia-reperfusion injury (Figure 1).

## 4. Renal Perfusion and Preservation Solutions

Renal perfusion and preservation solutions are meant to minimize ischemia-reperfusion injury by slowing down catabolic cell processes at a reduced temperature of 4–6 °C. They are to minimize the effects of anoxic hypothermia during storage and later during reimplantation. In recent years, there has been an increase in the number of studies focused on the development of an optimal solution that would improve the life functions of grafts and extend their storage period. Most optimizations are based on commercial solutions routinely used to store grafts prior to their transplantation. Table 1 shows the compositions of UW (Viaspan, University of Wisconsin), HTK (histidine-tryptophan-ketoglutarate), Biolasol, Euro-Collins, Belzer MPS and Vasosol solutions recommended for kidney perfusion and preservation, which have been subjected to hormone modification [4,23,24,25]. The solution compositions are based on pharmaceuticals that perform specific pharmacological functions. Phosphates, glucose, ribose, dextrose, adenine and adenosine are substrates of adenosine 5′-triphosphate (ATP) resynthesis. Histidine, phosphate, bicarbonate and HEPES buffers minimize the risk of metabolic acidosis and maintain isohydria in water spaces. Allopurinol inhibits xanthine oxidase activity. Lactobionate, raffinose, colloids (HES, pentastarch, Dextran 70) prevent cell swelling. Allopurinol, glutathione, mannitol, tryptophan and *N*-acetylcysteine neutralize reactive oxygen species (ROS). Citrate has anti-coagulant properties. Disodium edetate is a complexing agent for multivalent metal cations. Magnesium fumarate protects the physicochemical parameters of solutions and ensures their stability. l-glutamine stabilizes the cell membrane. l-arginine and nitroglycerine are nitric oxide precursors/donors responsible for vasodilation. The ionic composition of solutions is to minimize the effect of redistribution of ions in cells [2,26]. According to the hypothesis of Southard et al., solution effectiveness is based on the comprehensive, synergistic action of all substances that are part of it and show protective effects [27].

The UW solution can be used for mechanical organ perfusion and for static perfusion. UW proved to be ineffective in storing marginal organs [28], therefore IGL-1 solution was developed based on its composition. The risk of cardiovascular complications was reduced, among others, as a result of changing the concentration of K^+^ versus Na^+^ ions (UW: K^+^/125 mmol/L, Na^+^/25 mmol/L; IGL-1: K^+^/25 mmol/L, Na^+^/120 mmol/L) [2]. HTK to be effective in the storage of the liver, kidney, pancreas and heart. The solution is designed to counteract the retention of sodium and calcium ions in the intracellular space and buffer the extracellular space by means of the histidine/histidine HCl system during the ischemic period of the organ. The name of the solution comes from the three components of the formulation: histidine-tryptophan-ketoglutarate. Histidine is a natural buffer well tolerated by the body. It has a high penetration ability from the intravascular space into the interstitial space and exhibits low intracellular penetration, which counteracts cell swelling. Tryptophan is a stabilizer of the cell membrane and acts as an antioxidant through indirect metabolites of the kynurenine pathway. In turn, α-ketoglutarate is the substrate of anaerobic metabolism [23]. Biolasol is a solution developed in Poland for ex vivo perfusion and preservation of kidney, liver, pancreas and heart. The maximum storage time for organs in this solution is 24 h. Biolasol supports the structural and functional integrity of grafts and minimizes ischemia-reperfusion injury. The solution contains electrolytes, osmotically and oncotically active substances, buffering systems, substances preventing cellular acidosis, which are a source of energy, and antioxidants [4].

## 5. Hormones with a Potential Nephroprotective Effect

The kidney is an organ with relatively low regenerative capacity [29]. Its cells show limited ability to proliferate compared to cells of other parenchymal organs, e.g., the liver [30]. This is mainly due to the complexity of its structure, in which the cells are organized into functional compartments. The regeneration of individual nephrons does not correlate with the restoration of normal vital functions of the entire organ. The regenerative potential of the kidneys results mainly from the dedifferentiation of epithelial cells and their proliferation. The repair process is mainly stimulated by proteins with cytoprotective activity and growth factors released by cells [31,32]. This section discusses hormones with a potential nephroprotective effect, which were used to modify the compositions of commercial solutions intended for kidney perfusion and preservation. The obtained data are summarized in Table 2 and Table 3.

### 5.1. Melatonin

Melatonin is secreted in the pineal gland and extra-pineally, and shows multidirectional effects. It demonstrates the ability to bind to intracellular proteins, receptors in the cell nucleus membrane (ROR/RZR, retinoid orphan receptors/retinoid Z receptors), and receptors located in the mitochondrial and cell membrane [37]. The presence of high-affinity melatonin receptors MT1 and MT3 in the kidneys has been confirmed. The MT1 receptor (belonging to the GPCR/G protein coupled receptor subgroup) is coupled to G proteins, and its effect is a decrease in intracellular cAMP, and consequently a decrease in protein kinase A activity and CREB (cyclic-AMP response element-binding) phosphorylation and an increase in intracellular concentration of calcium ions [37,38,39]. It has been suggested that the inhibitory effect of melatonin on QR2 may have a protective effect in renal ischemia [40,41]. These processes affect cell proliferation.

Melatonin has a protective effect on the kidneys during ischemia-reperfusion. During neutralization of harmful reagents and stimulation of the production of antioxidant enzymes, metabolites are formed that increase the effectiveness of melatonin in inhibiting antioxidative stress (cyclic 3-hydroxymelatonin, *N*’-acetyl-*N*”-formyl-5-methoxykynuramine and *N*’-acetyl-5-methoxykynuramine) [34]. It stimulates the synthesis of reduced glutathione (GSH) [42,43]. It reduces electron leakage at the mitochondrial level [44]. Located on the surface of cell membranes, it protects them from oxidation, and by affecting their fluidity, it effectively removes free radicals before they damage the lipids and proteins of cell membranes [45].

Aslaner et al. [46] analysed the effect of melatonin added to the University of Wisconsin (UW) solution on the isolated rat kidney function after 2-, 24-, 36- and 48-h storage. They tested the effectiveness of the modified formulation based on the analysis of lactate dehydrogenase (LDH) and malondialdehyde (MDA) concentrations, as well as histological examinations. They observed the protective effect of the hormone after 36-h storage. In turn, after 48 h of preservation, the LDH level in UW + MEL perfusate samples was by 17% lower compared to UW perfusate samples. A similar relationship was observed when determining MDA. The concentration of this marker in UW + MEL perfusate samples was by 14% lower than in UW samples. Histological analysis confirmed the protective effect of the modified University of Wisconsin solution on kidneys during cold ischemia time. The authors suggest that melatonin as a component of preservation solutions can effectively extend the time of cold renal storage up to 48 h. However, it is important to use it at physiological levels.

### 5.2. Prolactin (PRL)

Prolactin is a hormone secreted by lactotropic cells of the anterior pituitary gland. Prolactin receptors belong to the class 1 cytokine receptor superfamily. They have the form of transmembrane peptide chains consisting of extracellular, transmembrane and cytoplasmic fragments. The distribution of PRL-R density in tissues is subject to hormonal regulation [47,49,52,53]. There are three prolactin receptor isoforms in humans, i.e., short (288aa), intermediate (376aa) and long (598aa), which shows the highest activity in transduction signal transmission [47]. Prolactin affects kidney function. It inhibits the activity of the sodium-potassium pump (Na^+^/K^+^ ATPase) in the proximal segment of the renal tubule [51,54]. It stimulates ENaC (epithelial sodium channels) sodium channel activity in renal epithelial cells [55]. It affects post-glomerular blood flow, which in consequence can lead to a decrease in reabsorption of sodium and water [56].

Caban et al. [57] modified the commercial HTK solution with PRL at doses of 0.2 mg/dL, 0.02 mg/dL and 0.01 mg/dL, then tested its efficacy in rinsing isolated porcine kidneys by simple hypothermia. They analysed standard parameters of normal renal function, i.e., LDH, AST, ALT activity, concentration of lactates, proteins, K^+^ and Ca^2+^ ions. Samples were collected after 24 and 48 h of cold ischemia. After 24 h, they observed significantly lower levels of LDH, AST and K^+^ compared to the control sample (no effect after 48 h). The authors suggested a cytoprotective effect of PRL at a dose of 0.02 mg/dL. It affects the reduction of tissue damage and the maintenance of normal metabolic pathways. In the following years, research into the effectiveness of PRL as a preservation solution component was continued. Biolasol was chosen as the model test solution. Ryszka et al. [58] confirmed the efficacy of rh-PRL at a dose of 1 µg/L in an isolated porcine kidney model. Compared to the control sample, they found less kidney oedema, maintenance of sodium potassium pump performance and cell membrane continuity. They observed no rapid increase in K^+^ concentration in perfundates during reperfusion and a decrease in ALT, AST and LDH activity after preservation. Ostróżka-Cieślik et al. [4], based on the same research model, analysed the impact of the synergistic action of PRL (1 μg/L) and vitamin C (0.088 g/L) added to Biolasol on the efficiency of isolated porcine kidney storage. They confirmed the effectiveness of pharmaceuticals used for modification in maintaining the structural and functional integrity of grafts.

### 5.3. Thyrotrophic Hormone (TSH)

Thyrotrophic hormone (TSH, thyroid-stimulating hormone) is a heterodimeric glycoprotein with a molecular weight of 28 kDa, composed of two subunits: α (encoded by the gene on chromosome 6) and specific β (encoded by the gene on chromosome 1). TSH receptors (TSHR, thyrotropin-secreting hormone receptors) belong to membrane receptors acting through the class A G protein complex (GPCR, G protein-coupled receptors) [65,66]. It has been found that the cell energy deficit correlates with a decrease in TSH synthesis and secretion [77,78].

Numerous studies confirmed that hypothyroidism increases the risk of chronic kidney disease (CKD) by impairing the glomerular filtration rate (GFR) [79,80]. This is due to the effect of thyroid hormones on their metabolism. Sellitti et al. suggested that the renal cortex shows the ability to express TSHR and thyroglobulin (Tg), which affects their dysfunction in thyroid diseases [73]. Duranton et al. have observed that rhTSH administered to patients with normal thyroid secretory function (euthyroid) improves their kidney function. The glomerular filtration rate (GFR) has also improved [81]. Basu et al. have confirmed that TSH increases GFR and increases renal blood flow (RBF) [82].

### 5.4. Corticotropin (ACTH)

Corticotropin is a peptide hormone consisting of 39 amino acids, secreted by corticotropic cells of the anterior pituitary gland. It is a trophic hormone of the banded and reticular adrenal layers which controls steroidogenesis. It indirectly affects the body’s carbohydrate, protein, lipid and water-electrolyte balance. It takes part in maintaining the homeostasis of the neuroimmunoendocrine system [83,84,85,86]. ACTH is a physiological agonist of the melanocortin system, which affects cell proliferation and maintenance of normal cell homeostasis. It has anti-inflammatory and immunomodulatory effects [87]. In addition, corticotropin exhibits antiproteinuric, lipid-lowering and renoprotective properties [85]. The ACTH receptor (ACTHR, melanocortin receptor 2 or MC2R) is a kind of melanocortin type 2 receptor. The receptor activates G proteins located in the outer plasma membrane [88,89]. ACTH receptors are located in the zona fasciculata of the human adrenal cortex, in the skin, in both white and brown adipocytes [90].

Si et al. analysed the impact of ACTH in a rat model of acute kidney injury (AKI), induced by the tumour necrosis factor (TNF). They have found that ACTH has a protective effect on the kidneys. It alleviates acute tubular necrosis, restores cell viability and inhibits apoptosis [87]. The use of ACTH in therapy eliminates proteinuria in patients with nephrotic syndrome [91,92].

Caban et al. [93] analysed the effectiveness of HTK modified with the addition of thyrotropin (1 μg/dL) and corticotropin (1 μg/dL), respectively. Isolated porcine kidneys were stored for 24 and 48 h by simple hypothermia in HTK, HTK+TSH and HTK+ACTH solutions. They found a beneficial effect of both hormones on the decrease in protein concentration, lactate concentration and pH in the collected perfusates after 48-h storage. The results obtained suggest the maintenance of a favourable metabolic effect in the graft (less protein depletion and reduced anaerobic metabolism). Activation of TSH and ACTH receptors in the kidney probably affects the maintenance of stabilization of the metabolic rate and adequate energy potential during 48 h of ischemia. The authors suggest that both hormones have an effect on decreasing neutrophil chemotaxis and nitric oxide synthesis.

### 5.5. Prostaglandin E-1 (PGE1)

Prostaglandin E-1 (PGE1) is a hormone-like substances produced by almost all body cells. It exhibits autocrine and paracrine effects by acting through membrane prostanoid spanning G protein-coupled receptors [94]. Four types of PGE1 receptors have been identified in different tissues and organs: EP1, EP2, EP3 and EP4. They are coded by different genes, have different expression regulation mechanisms and signal transduction pathways. Activation of the EP3 receptor (so-called “inhibitory”) reduces the level of cyclic adenosine monophosphate (cAMP), whereas the EP2 and EP4 receptors (so-called “relaxing”) increase the level of cAMP [95,96]. PGE1 has strong vasodilatory, anti-inflammatory, anti-proliferative properties, is a strong platelet aggregation inhibitor, stimulates smooth muscles, reduces platelet hyperactivity and thromboxane A2 (TXA2) level, reduces free Ca^2+^ levels in vascular smooth myocytes [97,98]. It has been found that PGE1 influences the maintenance of renal hemodynamic homeostasis by regulating blood flow and distribution as well as electrolyte and water excretion [99]. It shows anti-ischemic and tissue-protective abilities [100,101]. It improves the biochemical, oxidative and structural parameters of the kidneys during the ischemia-reperfusion (IR) period. In the case of renal artery stenosis, it prevents tissue contraction and inhibits a decrease in the glomerular filtration rate (GFR) [101,102]. PGE1 exhibits a stabilizing effect on endothelial and smooth muscle cell membranes during hypothermic preservation [103].

Polyak et al. [103] analysed the effect of prostaglandin E1 added to Belzer II solution during machine preservation (MP) or added to the University of Wisconsin solution during cold storage. Expanded criteria donors (ECD) stayed in The New York Presbyterian Hospital–Cornell Medical Centre. The kidneys (*n* = 150) were preserved by continuous hypothermic pulsatile perfusion (using 1 litre of Belzer II solution) at 4 °C and 60 beats per minute, at constant pressure. The other kidneys (*n* = 125) were preserved by cold storage (CS) for 12 h (4 °C). The addition of PGE1 to Belzer II increased renal flow, decreased renal resistance and reduced cellular calcium extrusion into the perfusate. The addition of PGE1 to the UW solution did not influence early graft function. The authors suggest that prostaglandin as a component of preservation solutions may supplement PGE1 lost during the period of cold ischemia and constitute the substrate necessary for proper graft functioning during the reperfusion period. PGE1 in combination with machine preservation significantly improves graft function compared to cold storage.

Guarrera et al. [104] compared the effectiveness of storing human kidneys in Vasosol and Belzer solutions, which are recommended for use by pulsed machine perfusion (MP). Prostaglandin E1 is present in the standard composition of Vasosol (VSL), but its dose was not specified by the authors. The kidneys were washed out of the blood and stored in the UW solution at 4 °C. Then VSL MPS or Belzer MPS were randomly allocated to MP. The kidneys were perfused en bloc at 4 °C to 6 °C at 60 beats/min with 1 L of perfusate. The perfusion pressure was below 60 mm Hg. PGE1 has been found to improve renal function parameters, including creatinine levels. Kidneys stored in E1-Vasosol had a higher survival rate than kidneys stored in UW (80.5% vs. 66.3%). Delayed graft function was significantly lower in the Vasosol E-1 group compared to the control group (12.2% vs. 21.2%).

Further studies by Polyak et al. [105] concerned the effectiveness of Vasosol compared to the UW solution. The dog kidney autotransplantation model was used. The grafts were washed and stored in the solutions for 24 h at 4 °C by cold ischemia. Based on the analysis of renal function parameters, Vasosol was found to provide optimal storage of grafts. Decreased levels of serum creatinine, blood urea nitrogen and tissue myeloperoxidase concentration were observed. The same authors [106] in another study found mild ultrastructural disruptions (slight cellular membrane condensation) in the graft perfusion in Vasosol (vs. 0.9% NaCl). They also confirmed the reduced levels of serum creatinine and blood urea nitrogen. The authors suggest that Vasosol is highly effective in improving kidney function in the early post-transplant period.

## 6. Trophic Factors with a Potential Nephroprotective Effect

### 6.1. Tumour Growth Factor β (TGF-β)

Successful kidney regeneration requires the activation of genes that regulate the growth and release of growth factors. Nephron proliferation is primarily affected by TGF-β (tumour growth factor β). TGF-β is a multifunctional cytokine that is involved in cell proliferation, differentiation and apoptosis, as well as in the synthesis of cell matrix components. It regulates the production of antibodies and induces other cytokines (IFNγ/interferon gamma, TNF-α/tumour necrosis factor α), and also stimulates the processes of angiogenesis and hematopoiesis [107,108,109,110,111]. Three TGF-β isoforms have been identified in mammalian cells, i.e., TGF-β_1_, TGF-β_2_, TGF-β_3_, with similar structure (two subunits connected by a disulphide bond), with similar biological functions and encoded by different genes. TGF-β affects cells through three classes of receptors: TβRI and ALK (activin receptor like kinase), TβRII (involved in signal transmission to the inside of the cell) and TβRIII (co-receptor without enzymatic activity). The receptors are located on the surface of cell membranes of all types of tissues [112,113,114,115]. A correlation was observed between chronic graft rejection and TGF-β-induced graft fibrosis processes. It is suggested that TGF-β may have an effect on prolonged and deep immunosuppression after allogeneic hematopoietic cell transplantation [113,114,115].

Kwon et al. [116] used an in vitro model for early detection of apoptotic changes resulting from cold ischemia. Mitochondrial membrane potential was determined by fluorescence intensity in primary canine kidney tubule cells. The obtained results suggest that the trophic factors (TFs) added to the UW solution affects the maintenance of normal mitochondrial functions and minimizes the risk of early apoptotic changes in vascular endothelial cells. It was found that mitochondrial membrane potential in human umbilical vein endothelial cells stored in TF-UW increased by 15%. TFS suppressed caspase 3 enzyme activity and activation in human umbilical vein endothelial cells. In an experiment carried out a year later [117], the same authors analysed the effect of UW+TF on the phosphorylation of signalling molecules ERK (extracellular regulated-signalling kinase) 1/2 and p38 MAPK (mitogen activated protein kinases) and of HO-1 (hemeoxygenase-1). The effectiveness of the modified formulation was tested by Western blotting in cells stored under cold ischemic conditions. Primary cultures of canine kidney proximal tubule cells (CKPTC) and human umbilical vein endothelial cells (HUVEC) were used in the experiment. The authors stated that the proposed trophic factors system limits ERK 1/2 and p38 MAPK activity induced by cold ischemic injury and increases HO-1 phosphorylation. TF reduce chronic injury in stored vessels, which improves kidney survival.

### 6.2. Nerve Growth Factor β (NGF-β)

NGF-β belongs to neurotrophins (with a molecular weight of 26 kDa) stimulating the growth and differentiation of peripheral and central neurons. It influences the normal morphology and function of neurons and affects the endocrine and immune systems. NGF-β acts on cells via NTRK1 membrane receptors (molecular weight of 140 kDa, it shows high affinity to tyrosine kinase) and TNFRSF1B (molecular weight of 75 kDa, it shows low affinity to the tumour necrosis factor receptor TNFR) [118,119,120,121,122,123]. It also affects cells outside the nervous system, i.e., mast cells, releasing from them mediators of the inflammatory process, eyeball cells, skin cells, lymphocytes. NGF-β and its receptors are involved in the regulation of responses to tissue damage and inflammation [124,125].

Waller et al. [126] modified the UW solution with the addition of trophic factors (TFs: NGF β/20 µg/L, SP/2.5 µg/L, IGF-1/10 µg/L). The tests were performed in vitro using the model of canine kidney tubule cells. The cells were stored in UW or UW with TFs for 3 days at 0–2 °C. The content of H_2_O_2_ was measured at 15-min intervals starting at 0 min and concluding at 90 min of warm reperfusion. TFs reduce the level of H_2_O_2_, which indicates that they mediate the reduction in free radical (ROS) secretion during the cold ischemia period. TFs help reduce the level of oxidative stress in cells during storage and reperfusion. This may be due to the impact of trophic factors on protein stability (or expression) in an antioxidant system or the impact on mitochondrial protection. In addition, cell viability/cytotoxicity was evaluated after 3 days of cold storage. It was confirmed that trophic factor supplementation increased cell viability.

### 6.3. Epidermal Growth Factor (EGF)

EGF is made of 53 amino acids (6 of them are cysteine residues determining EGF bioactivity) and has a molecular weight of 6 kDa. It participates in the processes of cell proliferation, determination, differentiation, migration, apoptosis and DNA repair. It is involved in the repair processes of mature and damaged organs [127,128,129,130]. The EGFR receptor (Human Epidermal Receptors, ErbB1, HER1) is a transmembrane protein with a molecular weight of 170 kDa, which exhibits tyrosine kinase activity [129,131]. In its structure, the following domains can be distinguished: intracellular, transmembrane and extracellular [130]. EGFR is secreted in most cell types, except hematopoietic cells. The proper activity of EGFR determines the maintenance of skin cell homeostasis and the proliferative balance of epidermal cells [132].

McAnulty et al. [137] used a canine kidney autotransplantation model. They analysed the effectiveness of the modified solution based on the measurement of creatinine in the blood serum of animals after transplantation. The storage time of kidneys by simple hypothermia (excluding perfusion) until implantation into recipients was 3–6 days. Creatinine levels were found to decrease with increasing kidney storage time. The lowest creatinine concentration was found in dogs whose kidneys were stored in the modified UW solution for 6 days. In dogs whose kidneys were stored for 4 days in TFS-UW, the mean serum creatinine level was 2.9 ± 0.2 mg/dL and reached the physiological value after 6 days. In turn, kidney storage in the original UW solution for 3 days resulted in higher creatinine values: 4.2 ± 0.3 mg/dL in the serum of dogs, and a longer recovery time for this parameter to the physiological value, i.e., 14 days. UW supplementation with TFS improves the quality of stored kidneys and extends the time of their effective hypothermic preservation. The authors suggest that many cell signalling pathways retain their activity at low temperatures and may interact with trophic factors. Pharmaceuticals used for UW supplementation exhibit synergistic effects. However, the authors doubt whether the use of all trophic factors components was necessary. However, the lack of TFS in the preservation solution exacerbates ischemic damage.

### 6.4. Insulin-Like Growth Factor-1 (IGF-1)

IGF-1 is a polypeptide hormone with a molecular weight of 7.65 kDa and a structure similar to proinsulin [138,139]. 95% of IGF-1 is present in plasma as a complex with IGFBP-3 (insulin-like growth factor binding protein-3) and ALS (acid-labile subunit) [142]. IGF-1 secretion depends on the mammalian species, sex, age, circadian rhythm, genetic factors and ongoing disease processes [143]. Two types of IGF-1 receptors were identified, i.e., IGF-1R (insulin-like growth factor-1 receptor) showing tyrosine kinase activity and located on cell membranes, and IGF-2R (insulin-like growth factor-2 receptor). The activation of the immune system in the course of multi-organ damage reduces IGF-1 concentration in the blood [143,146]. An increase in IGF-1-induced proliferation was observed in kidney cells, thyroid cells, uterine epithelial cells, keratinocytes, osteoblasts, smooth muscle and skeletal muscle cells and chondrocytes [147]. Chronic renal failure correlates with reduced bioavailability of IGF-1 [148]. It is also suggested that an increase in IGF secretion in renal tubule epithelial cells protects against proliferation of damaged cells in the course of acute kidney injury (AKI) [149].

Petrinec et al. conducted studies on the effectiveness of IGF-1 in the initiated renal injury in a canine autotransplantation model of delayed graft function. The dogs underwent unilateral nephrectomy. IGF-1 was added to the Euro-Collins solution at a dose of 10^−7^ mol/L and its effectiveness in renal storage was assessed in relation to Euro-Collins supplemented with the addition of acetic acid. The grafts were stored in the test solutions for 24h (4 °C). The degree of kidney damage was evaluated within 5 days after contralateral nephrectomy and autotransplantation. Lower creatinine (3.5 ± 0.3 versus 6.9 ± 1.9 mg/dL) and urea levels (68 ± 4 versus 101 ± 15 mg/dL) in the blood were found in the dogs that received kidneys rinsed with Euro-Collins with the addition of IGF-1compared to the control group. Inulin clearance was twice as large (1.37 ± 0.16 mL/min/kg versus 0.77 ± 0.13 mL/min/kg; *p* < 0.05). Histopathological features were more optimal. The authors suggest that IGF-1 may be applicable to acute renal injury in cadaveric renal transplantation [150].

### 6.5. Hepatocyte Growth Factor (HGF)

Hepatocyte growth factor (HGF) belongs to plasminogen proteins and is composed of two subunits: α (69 kDa) and β (34 kDa) connected by a disulphide bond. It participates in the process of DNA synthesis in hepatocytes, regulates the physiological development of the liver and participates in its regeneration. HGF binds to its c-Met receptor on epithelial and endothelial cells [151,152]. It has been found to exert a protective effect on epithelial and non-epithelial organs (heart, brain) by means of anti-apoptotic and anti-inflammatory signals [153]. An increase in HGF secretion is observed during the IR period [154]. Exogenous HGF induces the growth of regenerating tubular cells in the kidney after unilateral nephrectomy. Endogenous HGF, in turn, participates in maintaining renal homeostasis. The administration of rh-HGF in acute renal failure affects tubular regeneration [153].

Nakatani et al. [155] studied the effectiveness of hepatocyte growth factor (HGF) based on the ischemic canine kidney model. Kidneys were rinsed with Euro-Collins with HGF for 4 h (4 °C). They found that it accelerated both the recovery of renal blood flow (RBF) and the glomerular filtration rate (GFR). Intrarenal arterial infusion of HGF in a normal canine kidney had no effects on renal hemodynamics. It is likely that HGF contained in Euro-Collins protects vascular endothelium. It regulates the activity of endothelin-1 and endothelial nitric oxide synthase. The authors suggest that HGF-Euro-Collins can also counteract ischemic injury in human kidneys and improve graft survival.

### 6.6. Bovine Neutrophil Peptide

BNP-1 (bovine neutrophil peptide-1) is a peptide neurohormone produced by myocardial cardiomyocytes, mainly ventricular and to a lesser extent atrial ones [156]. It acts as a regulator of blood pressure and circulating blood volume. It inhibits the activity of the sympathetic nervous system, renin-angiotensin-aldosterone system, and inhibits fibrosis processes in the heart and blood vessels. It increases urinary excretion of water and Na^+^ ions as a result of increased glomerular filtration and reduced Na^+^ and water resorption in the distal renal tubules. The consequence of this process is the relaxation of the muscular membrane of blood vessels and reduction of peripheral vascular resistance. It also has antiproliferative effects [158,159]. The human gene encoding BNP was found on chromosome 1 (1p36.2) [160]. NPR-A/NPR1, NPR-B/NPR2 and NPR-C/NPR3 (natriuretic peptide receptor type A, B, C) show affinity to BNP [161,162,163]. The release of BNP from ischemic myocardium protects the endothelium, reduces peroxide radical (ROO·) synthesis, lysosome secretion, matrix metalloproteinase-9 (MMP-9) concentration, and inhibits neutrophil adhesion [164].

Intravenous BNP infusion reduces vascular resistance and blood pressure. Coronary and renal circulation vessels are particularly sensitive [165].

### 6.7. Substance P (SP)

SP has also been used to modify the solutions. It is a neuropeptide consisting of 10 amino acids and belonging to the tachykinin group [166,167]. SP occurs in the central nervous system (midbrain, hypothalamus, amygdala, striatum), in the peripheral nervous system (primary afferent fibres), in the cells of the bone marrow stroma, endothelial cells, cells of the cardiovascular system, respiratory system, genitourinary tract, skin, muscles, salivary glands, thyroid, and eosinophils [168,169,170,171,172]. SP can act autocrinely or paracrinely. It affects the metabolic processes of nerve tissue, dilates vessels and increases vascular permeability, modulates the activity of the hematopoietic system, minimizes the effects of apoptosis-inducing compounds, modulates the immune response, activates macrophages to produce cytokines (TNF-α, IL-1), induces proliferation and differentiation of lymphocytes, regulates airway smooth muscle function, stimulates proliferation of epithelial cells of pleural tissue, regulates gastrointestinal motility and participates in pain neurotransmission [173,174,175,176,177,178]. It shows high affinity to the NK-1 receptor (neurokinin receptor) [179]. This receptor was found around the amygdala, hypothalamus, frontal lobes, in stem cells isolated from umbilical cord blood, in bone marrow, lymphocytes, thrombocytes, macrophages, monocytes, in the thymus, on the surface of intestinal epithelial cells, in the wall of submucosal vessels, in the vascular endothelium and at the nerve endings [180,181,182,183,184,185,186,187,188]. Under hypoxic conditions, SP has an anti-apoptotic effect [189].

It is difficult to clearly confirm the protective effect of hormones on the kidneys based on the above studies. However, considering the results of extensive research on the effectiveness of hormones in transplanting other organs, their use as a component of perfusion and preservation solutions is potentially promising for the development of new therapeutic strategies in renal transplantation.

## 7. Effectiveness of Hormone and Trophic Factors—Modified Solutions in Protecting Other Organs

There have been many studies on the effectiveness of hormones added to preservation solutions in protecting the liver and heart. Liver perfusion and preservation solutions were modified with the addition of melatonin (IGL-1, UW), prolactin (HTK), dopamine (HTK), erythropoietin (HTK), insulin (UW), glucagon (UW), relaxin (UW, HTK), prostaglandin E1 (HTK) and trophic factors (IGL-1, UW). Melatonin reduces transaminase levels, affects high bile production and high BSP clearance (sulfobromophthalein clearance). It has been found to increase NO induction through the activation of constitutive nitric oxide synthase (eNOS) and reduced vascular resistance. It lowers mitochondrial oxidative stress and increases respiratory chain activity. Prolactin reduces the number of released transaminases, lactate dehydrogenase, lactic acid, which suggests its ability to inhibit liver cell cytolysis. It affects the stabilization of cell membranes, reducing oncotic necrosis. Dopamine reduces aminotransferases, increases bile flow and reduces lipid peroxidation. Erythropoietin ameliorates I/R-associated endothelial denudation in steatotic livers. The addition of insulin intensifies ischemia-reperfusion injury. It lowers ATP, adenine nucleotide (TAN) pool and hepatocyte energy resources. Glucagon affects the maintenance of normal structural integrity of hepatocytes, increases bile production in the liver and regenerates ATP levels in tissues. Relaxin reduces the activity of malonyldialdehyde and myeloperoxidase. It affects decreased peroxidation and increased oxygen. Prostaglandin E1 reduces ALT activity and hyaluronic acid concentration. Trophic factors slow down the release of aminotransferases and increase bile production. They can affect the activity of AKT (serine-threonine kinase) and eNOS (endothelial nitric oxide synthase) and inhibit TNF-α proinflammatory cytokine release. Trophic factors can induce hepatocyte proliferation and increase DNA synthesis in hepatocytes [2,190].

Heart perfusion and preservation solutions were modified with the human recombinant hepatocyte growth factor/hrHGF (Euro-Collins), erythropoietin (Celsior) and melatonin (HTK, St. Thomas). HrHGF acts as an inhibitor of apoptosis, improves left ventricle functions. Erythropoietin activates the RISK pathway and inhibits apoptosis [191]. In the case of melatonin, test results are contradictory. Schaefer et al. suggest that melatonin did not improve the functional recovery during reperfusion of HTK protected hearts [191,192]. The results obtained by other authors indicate its cardioprotective effect. Melatonin added to the St. Thomas solution slows down the release of creatine kinase (CK), ensures high energy phosphate levels and improves histological parameters [193].

## 8. Conclusions

Kidney injury as a result of warm and cold ischemia as well as reperfusion is one of the factors determining proper graft function after transplantation. Minimizing these processes is an important and fascinating research topic in the aspect of searching for new therapeutic strategies. Given the progress in knowledge of the function of hormones in the regeneration of kidney cells, it is crucial to conduct further research towards understanding the mechanisms of their action. Despite the promising results of preclinical studies, it is difficult to conclude about their potential effectiveness in clinical trials. However, due to their confirmed nephroprotective effect, it can be expected that in the near future the research will be extended to other hormones (hormone systems), which will consolidate knowledge on the safety and effectiveness of their use and allow them to enter clinical trials. We suggest that placing two or more protein-peptide substances with different mechanisms of action in a preservation solution may enhance its nephroprotective potential. The synergistic effect of trophic factors in combination with MEL, PRL, TSH, ACTH and PGE1 may increase their effectiveness in the prevention and therapy of ischemia-reperfusion injury. Prolactin and growth hormone (GH) are similar in terms of structure and function. We suspect that their therapeutic use in the PRL/GH system may enhance their activity in cell proliferation and differentiation. We believe that hormones as components of preservation solutions are a forward-looking strategy for developing renal transplantation.

## Figures and Tables

**Table 1:** Composition of preservation solutions.

Component	Blood	UW	HTK	Biolasol	Euro-Collins	Belzer MPS	Vasosol
IC/EX	EX	IC	EX	EX	IC	EX	EX
Electrolytes (mmol/L)
K^+^	5	125	10	10	115	25	28
Na^+^	140	30	15	105	10	100	110
Ca^2+^	2.5	-	0.015	0.5	-	0.5	0.5
Mg^2+^	0.9–1.2	5	4	5	-	5	5
Cl^−^	103	20	32	10.5	15	1	1
SO_4_^2−^	0.5	5	-	-	-	-	-
Colloids (g/L)
HES	-	50	-	-	-	0.25	-
Pentastarch	-	-	-	-	-	-	50
Dextran 70	-	-	-	0.7	-	-	-
Albumine	42	-	-	-	-	-	-
Globuline	24	-	-	-	-	-	-
ROS scavengers (mmol/L)
Allopurinol	-	1	-	-	-	-	-
Glutathione	-	3	-	-	-	-	-
Mannitol	-	-	30	-	-	30	30
Tryptophan	-	-	2	-	-	-	-
*N*-acetylocysteine	-	-	-	-	-	-	70 [mg]
Buffers (mmol/L)
Histidine	-	-	198	-	-	-	-
HPO_4_^2-^/H_2_PO_4_^-^	1.12–1.45	25	-	-	58	25	25
HCO_3_^-^	27	-	-	5	10	-	-
HEPES	-	-	-	-	-	10	10
Impermeants (mmol/L)
Lactobionate	-	100	-	-	-	-	70
Raffinose	-	30	-	-	-	-	-
Citrate	-	-	-	30	-	-	-
Glucose	7	-	-	167	195	10	-
Gluconate	-	-	-	-	-	85	90
Ribose	-	-	-	-	-	5	5
Dextrose	-	-	-	-	-	10	10
Additives (mmol/L)
Adenine	-	-	-	-	-	5	5
Adenosine	-	5	-	-	-	-	-
l-Arginine	-	-	-	-	-	-	5 [mg]
Ketoglutarate	-	-	1	-	-	-	1 [mg]
EDTA	-	-	-	5	-	-	-
Fumarate	-	-	-	5	-	-	-
l-Glutamine	-	-	-	-	-	-	2
Nitroglycerin	-	-	-	-	-	-	5 [mg]
Prostaglandin E1	-	-	-	-	-	-	0.001 [mg]
pH	7.4	7.4	7.2	7.4	7.3	7.4	N/A
Viscosity (cP)	1.60	5.01	1.68	2.90	N/A	N/A	N/A
COP (mm Hg)	28 (36.6 °C)	31.9 (5 °C)	1.45 (5 °C)	N/A	N/A	N/A	N/A
Osmolality (mOsm/kg H_2_O)	308	320	310	330	406	300	300

IC—intracellular, EX—extracellular, HES—hydroxyethyl starch, COP—colloid osmotic pressure, EDTA—ethylenediaminetetraacetic acid, MPS—machine preservation solution.

**Table 2:** The effect of hormones and trophic factors on quality of organs.

Type Hormone /Trophic Factor	Chemical Class	Source	Mechanism of Hormone Action	Plasma/Serum Concentration	Circulating Half-Life	Distribution	Functions	Physiological Effects on the Organs	References
Melatonin	Amine	Pineal gland	Cyclic AMP	0–20 pg/mL during the day 20–100 pg/mLat night	Endogenous melatonin: 30–60 min Exogenous melatonin: 12–48 min	Cerebrospinal fluid, bile, follicular fluid, semen, amniotic fluid, preovulatory follicles, breast milk, renal	Sleep cycles; Multidirectional effects	Stabilizes cell membranes; Scavenges ROS; Regulates redox network; Prevention of apoptosis; Influence on immunological system;	[33,34,35,36,37,38,39,40,41,42,43,44,45,46]
Prolactin (PRL)	Peptide	Pituitary, anterior	Tyrosine kinase mechanism	women: 10–25 ng/mL men: 10–20 ng/mL	20 – 50 min	Central nervous system, adrenal glands, skin, bone tissue, lungs, heart, skeletal muscles, liver, salivary glands, pancreatic islets, gastrointestinal tract, kidneys (proximal kidney tubules, distal tubules, renal cortex tubules), bladder, lymphatic system, ovaries, fallopian tubes, mammary gland, uterine endometrium, placenta, foetal membranes, testes, epididymis, seminal vesicles	Stimulates lactation; Multidirectional effects	Neurotransmitter; Immunomodulator;Metabolism regulator; Prevention of apoptosis;	[47,48,49,50,51,52,53,54,55,56,57,58]
Thyrotropin (TSH)	Glycoprotein	Pituitary, anterior	Cyclic AMP	0.35–4.94 µU/mL	55 min	Thyroid follicular cells, osteoblasts, osteoclasts, adipose tissue, retro-orbital tissue, lymphocytes, thymus, pituitary, testes, kidney, brain, adipose cells, fibroblasts, heart, human skin	Stimulates synthesis and secretion of thyroid hormones	Immunomodulator; Regulatory effects on metabolic and inflammatory processes;	[59,60,61,62,63,64,65,66,67,68,69,70,71,72,73,74,75,76,77,78,79,80,81,82]
Corticotropin (ACTH)	Peptide	Pituitary, anterior	Cyclic AMP	<46 pg/mL	10 min	Adrenal cortex, skin, adipocytes	Stimulates synthesis and secretion of adrenal cortical hormones	Immunomodulator; Regulatory effects on metabolic and inflammatory processes;	[83,84,85,86,87,88,89,90,91,92,93]
Prostaglandin E-1	Lipid hormone-like molecules	Almost all body cells	Cyclic AMP	Endogenous < 0.25 ng/mL	42 s	Myometrium, pulmonary veins, colon, skin, mast cells, plasma membrane, leukocytes, smooth muscle, central nervous system, reproductive system, bones, cardiovascular system, kidney, urinary bladder, cell nuclei membranes	Anti-inflammatory role	Anti-inflammatory effect; Antiproliferative effect; Vasodilatory effect;	[94,95,96,97,98,99,100,101,102,103,104,105,106]
Tumour growth factor β (TGF-β)	Peptide	Platelets, most cell types	Serine kinase mechanism	<0.2 ng/mL	2–3 min	Fibroblasts, endothelial cells, keratinocytes, lymphocytes, monocytes	Involved in cell proliferation, differentiation and apoptosis	Regulates fibroblast activity; Regulates keratinocyte proliferation, Prevents immune mediated apoptosis by infiltrating lymphocytes; Promotes matrix synthesis; Regulates the production of antibodies; Stimulates the processes of angiogenesis and hematopoiesis;	[107,108,109,110,111,112,113,114,115,116,117]
Nerve growth factor β (NGF-β)	Peptide	A protein secreted by a neuron’s target tissue	Tyrosine kinase mechanism	0.05 pg/mL	5 min	Nervous system (including sympathetic ganglia), kidney, spleen, liver, salivary gland	Maintenance of sympathetic and sensory neurons	Influences the endocrine and immune systems; Regulates the response to tissue damage and inflammation;	[118,119,120,121,122,123,124,125,126]
Epidermal growth factor (EGF)	Peptide	Keratinocytes, macrophages	Tyrosine kinase mechanism	women: 604 pg/mL men: 780 pg/mL	8 min	Epithelium, endothelial cells, liver, thyroid	Involved in cell proliferation, differentiation and apoptosis	Regulates mesenchymal and epithelial cells proliferation;	[127,128,129,130,131,132,133,134,135,136,137]
Insulin-like growth factor-1 (IGF-1)	Peptide	Liver skeletal muscle, fibroblasts, macrophages	Tyrosine kinase mechanism	183–850 ng/mL	10 min	Growth cartilage, liver, kidney (proximal tubule cells), lungs, heart, testes	Pleiotropic effect	Regulates keratinocyte proliferation; Regulates fibroblast proliferation; Regulates endothelial cell activity; Stimulates tissue repair processes; Increases glycolysis; Reduces lipolysis; Stimulates the immune system; Stimulates cell enzyme systems;	[138,139,140,141,142,143,144,145,146,147,148,149,150]
Hepatocyte growth factor (HGF)	Peptide	Multiple cells	Tyrosine kinase mechanism	0.4–0.8 ng/mL	5 min	Fibroblasts, epithelial and endothelial cells, fat-accumulating cells in the liver, bone marrow stromal cells	Involved in cell proliferation, differentiation and apoptosis	Mediates angiogenesis; Mediates regeneration cells; Acts as a mitogen, motogen, and morphogen in many cells and tissues;	[151,152,153,154,155]
Bovine neutrophil peptide-1 (BNP-1)	Peptide	Myocardial cardiomyocytes	Cyclic BMP	<200 pg/mL	22 min	Blood vessels, endothelium, brain, eye, kidneys, adrenals, lungs, adipose tissue, smooth muscle cells	Regulates of blood pressure and circulating blood volume	Inhibits the activity of the sympathetic nervous system, renin-angiotensin-aldosterone system; Inhibits fibrosis processes in the heart and blood vessels; Increases urinary excretion of water and Na+ ions as a result of increased glomerular filtration and reduced Na+ and water resorption in the distal renal tubules; antiproliferative effects	[156,157,158,159,160,161,162,163,164,165]

AMP–adenylyl cyclase mechanism; GMP–guanylate cyclase mechanism.

**Table 3:** Strategies based on modifications of preservation solutions.

Author, Year of Publication	Hormone/Trophic Factors	Species	Preservation Solution Modification/Cold Ischemia	Outcome Measures, (Intervention, I/Control, C)	Hormone/Trophic Factors Dose	Drugs/Substances Used Simultaneously/Dose	Effects of Hormone/Trophic Factors
Aslaner et al. 2013 [46]	Melatonin	Rat	UW /2 h, 24 h, 36 h, 48 h, 4 °C/SCS	I: UW + MEL C1: Ringer Lactate C2: UW	30 mg/L	-	Prevented enzyme elevation Decreased lipid peroxidation Decreased level of MDA Prevented pathological kidney injury
Caban et al. 2010 [57]	Prolactin	Pig	HTK/24 h; 48 h, 4–6 °C/SCS	I: HTK + PRL C1: Ringer C2: HTK	0.2 mg/dL 0.02 mg/dL 0.01 mg/dL	-	Protective effect of PRL with the 0.02 mg/dL dose Lower AST, LDH and K^+^ after 24h No changes observed in 48h
Ryszka et al. 2016 [58]		Pig	Biolasol /24h, 4 °C/SCS	I: Biolasol + rh-PRL C: Biolasol	1 μg/L	-	Increased ALT, AST during perfusion and preservation Decreased ALT, AST during reperfusion Decreased pH and osmolarity Increased K^+^, decreased Na^+^
Ostróżka-Cieślik et al. 2018 [4]		Pig	Biolasol /48 h, 4 °C/SCS	I: Biolasol + vit.C + PRL C1: Biolasol C2: Biolasol + vit.C	1 μg/L	Vitamin C 0.088g/L	Lower levels of AST, ALT Maintenance of the normal cytoskeleton Synergistic effect of PRL with vitamin C
Caban et al. 2013 [93]	Thyrotropin	Pig	HTK /24 h and 48 h, 4 °C/SCS	I: HTK + TSH C1: Ringer C2: HTK	1 μg/dL	-	Lower protein concentrations Lower concentrations of AST Decreased anaerobic metabolism Stabilization of the metabolic rate
Caban et al. 2013 [93]	Corticotropin	Pig	HTK /24h and 48h, 4 °C/SCS	I: HTK + ACTH C1: Ringer C2: HTK	1 μg/dL	-	Lower protein concentrations Decreased anaerobic metabolism Stabilization of the metabolic rate
Polyak et al. 1999 [103]	Prostaglandin E-1	Human	Belzer/pulsatile perfusion, 4 °C, 60 beats/min with 1 L solution/MP; UW/ 12 h, 4 °C/SCS	I: Belzer + PGE-1 C: BelzerI: UW + PGE-1 C: UW	-	-	Increased renal flow (MP) Decreased renal resistance (MP) Reduced cellular calcium extrusion (MP) Improved early graft function in ECD kidneys (MP)
Guarrera et al. 2004 [104]		Human	Vasosol, /pulsatile perfusion 4–6 °C, 60 beats/min with 1 L solution/MP	I: VSL with PGE-1 C: Belzer	-	-	Decreased level of creatinine Improved early graft function
Polyak et al. 2008 [105]		Dog	Vasosol /24 h, 4 °C/SCS	I: VSLwith PGE-1 C: UW	0.001 mg	-	Decreased level of serum creatinine Decreased level of blood urea nitrogen Decreased level of tissue myeloperoxidase concentrations
Polyak et al. 2008 [106]		Dog	Vasosol /in situ	I: VSLwith PGE-1 C: 0.9% NaCl	0.001 mg		Decreased level of serum creatinine Decreased level of blood urea nitrogen Mild ultrastructural disruptions Improved early graft function
Petrinec et al. 1996 [150]	Trophic Factors	Dog	Euro-Collins /24h, 4 °C/SCS	I: Euro − Collins + IGF-1 C: Euro − Collins + acetic acid	IGF-1: 10^−7^ mol/L	-	Lower serum creatinine levels Lower blood urea nitrogen levels Inulin clearance was greater Improved histologic characteristics Improved post autotransplant kidney function (at 5 days)
McAnulty et al. 2002 [137]		Dog	UW /4 days, 4 °C/SCS	I: UW + TF C: UW	NGF β: 20 µg/L EGF: 10µg/L SP: 2.5 mg/L IGF-1: 10 µg/L BNP-1: 1 mg/L	-	Prolongation of storage duration (up to 6 days) Improved post transplant kidney function
Waller et al. 2007 [126]		CKPTC	UW /3 days, 0–2 °C/SCS	I: UW + TF C: UW	NGF β: 20 µg/L SP: 2.5 µg/L IGF-1: 10 µg/L	Bactenecin: 1 mg/L	Decreased content of H_2_O_2_ Reduced free radical production Increased cell viability after recovery from cold ischemic storage
Nakatani et al. 2002 [155]		Dog	Euro-Collins /3h, 4 °C/SCS	I: Euro − Collins + HGF C: HGF/arterial infusion	HGF: 60 µg	-	Accelerated recovery of renal blood flow Accelerated glomerular filtration rate
Kwon et al. 2007 [116]		MDCK	UW /4 days, 4 °C/SCS	I: UW + TF C: UW	TGF β: 20 µg/L SP: 2.5 µg/L IGF- 1: 10 µg/L BNP-1: 1 µg/L	-	Protected mitochondrial function Suppressed caspase 3 enzyme activity Reduced early apoptotic changes
Kwon et al. 2008 [117]		CKPTC	UW /4 days, 4 °C/SCS	I: UW + TF C: UW	NGF: 20 µg/L SP: 2.5 µg/L, IGF-1: 10 µg/L BNP-1: 1 µg/L	-	Decreased ERK 1 and 2 activity Limited ERK 1/2 and p38 MAPK Restricted increases in HO-1 phosphorylation

MDA: Malondialdehyde; RL: Ringer Lactate; UW: University of Wisconsin; MEL: Melatonin; PRL: prolactin; rh-PRL: recombinant human prolactin; LDH: lactate dehydrogenase; AST: aspartate aminotransferase; ALT: alanine aminotransferase; HTK: histidine-tryptophan-ketoglutarate solution; UW: University of Wisconsin solution; TSH: thyrotropin; ACTH: corticotrophin; MDCK: Madin-Darby canine kidney; TF: trophic factors; CKPTC: canine kidney proximal tubule cells; NGF: nerve growth factor; IGF-1: insulin-like growth factor-1; BNP-1: bovine neutrophil peptide-1; TGF-β: growth factor β; SP: substance P; EGF: epidermal growth factor; ERK: extracellular regulated-signalling kinase; MAPK: mitogen activated protein kinases; HUVEC: human umbilical vein endothelial cells; PGE-1, Prostaglandin E-1; MP, machine preservation; CS, static cold storage; ECD, expanded criteria donor; VSL, Vasosol solution; HGF, hepatocyte growth factor, SCS, simple cold storage.
